# Aseptic Meningitis Secondary to Antibiotic Therapy

**DOI:** 10.7759/cureus.14454

**Published:** 2021-04-13

**Authors:** Juan A Negron-Diaz, Linda C Klumpp, Cynthia A Mayer, Jeffrey Jordan

**Affiliations:** 1 Internal Medicine, Citrus Memorial Hospital, Inverness, USA; 2 Infectious Disease, Citrus Memorial Hospital, Inverness, USA

**Keywords:** aseptic meningitis, drug-induced aseptic meningitis, viral meningitis, tmp-smx, bactrim

## Abstract

Drug-induced aseptic meningitis is a rare entity. Diagnosis of drug-induced aseptic meningitis can be challenging due to the difficulty in distinguishing clinical presentation from bacterial or viral meningitis. We present a case of a 52-year-old Caucasian female patient who presented to the emergency room on two different occasions with severe headache, neck pain, and confusion. Initial cerebrospinal fluid (CSF) analysis showed lymphocytic pleocytosis, and empirical intravenous acyclovir was initiated. Bacterial and viral CSF analysis and cultures were negative. The patient completely recovered. Several days later, the patient returned to the emergency room with similar symptoms. Second CSF analysis revealed neutrophilic pleocytosis, and empirical intravenous antibiotic and antiviral therapy were started. Bacterial, fungal, and viral CSF analysis and cultures were negative. Imaging studies of the brain were unremarkable on both occasions. The patient reported taking trimethoprim-sulfamethoxazole (TMP-SMX) for right foot infection before and after the initial presentation. The patient's symptoms resolved without neurological sequelae after discontinuation of TMP-SMX. This case report highlights the importance of taking a detailed history to diagnose drug-induced aseptic meningitis.

## Introduction

Aseptic meningitis is a presentation of meningeal inflammation with clinical and laboratory findings without evidence of bacterial infection confirmed by cerebrospinal fluid (CSF) culture. The most common cause is enteroviruses, however, less common causes include autoimmune disease, leukemia, vaccines, and drugs [1]. Drug-induced aseptic meningitis (DIAM) is a rare adverse reaction believed to have an immune-hypersensitivity nature affecting the central nervous system [2]. Many drugs have been implicated, yet non-steroidal anti-inflammatory drugs (NSAIDs) are most commonly involved [3]. Antibiotics, immune-modulatory agents, intrathecal steroids, and antiepileptics have also been associated with DIAM [4, 5]. Among the antibiotics, trimethoprim-sulfamethoxazole (TMP-SMX) is more frequently linked to DIAM even though many other antimicrobial agents have also been reported [6, 7]. Diagnosis can be challenging due to the difficulty in distinguishing DIAM from bacterial or viral meningitis.

## Case presentation

A 52-year-old Caucasian female patient presented to the emergency room (ER) on two different occasions due to severe headaches and altered mental status. The patient has a known past medical history of chronic lumbar pain secondary to herniated discs with multiple lumbar surgeries and tobacco use. Initially, the patient presented to the ER eight days earlier complaining of a one-day history of headaches, chest pain, palpitations, and confusion. Family reported that the patient was experiencing short-term memory loss and confusion. Home medications upon admission were methadone for chronic pain management and trimethoprim-sulfamethoxazole (TMP-SMX), which was prescribed due to an ulcer between the right fourth and fifth toes 17 days prior to the initial presentation to the ER visit. A wound culture obtained during that visit to the ER grew methicillin-sensitive *Staphylococcus aureus.*

Initial physical examination demonstrated an alert but confused and distressed patient. Vital signs: oral temperature of 97.2°F, pulse of 98 beats per minute (bpm), respiratory rate of 20 per minute (pm), blood pressure 110/80 mmHg, and oxygen saturation 100% on room air. Laboratory values were white blood count (WBC) 11.1 K/mcL, hemoglobin (Hgb) 12.8 g/dL, hematocrit (Hct) 39.3%, and creatinine 0.8 mg/dL. Urine drug screening (UDS) was positive for methadone and urine analysis (UA) was normal. Electrocardiogram (EKG) rhythm was consistent with sinus tachycardia. Computed tomography (CT) of the brain showed normal soft tissue structures and calvarium without intracranial pathologies. Magnetic resonance imaging (MRI) of the brain showed normal findings without evidence of ischemia or hemorrhage along with normal bony and soft structures of the upper cervical spine. TMP-SMX was held upon admission. 

The cerebrospinal fluid (CSF) analysis revealed WBC of 13/cm³ with lymphocyte predominance, lymphocyte 92%, neutrophils 8%, red cell count 0/cm³, glucose 55 mg/dL, and protein 41 mg/dL. Results suggested viral meningitis. The patient was started on empirical intravenous (IV) acyclovir. CSF analysis for Herpes Simplex Virus (HSV) 1 & 2 polymerase chain reaction (PCR), West Nile Virus IgM/IgG, and Cryptococcal antigen were negative and culture did not yield bacterial or fungal growth. Venereal Disease Research Laboratory test (VDRL) was non-reactive. CSF cytology revealed monocytes and lymphocytes, but it was negative for malignancy. CSF Gram stain smear was negative. On the second day of admission, the patient developed a temperature of 103.2°F but no further febrile episodes were reported later during her hospitalization. The patient continued to present with confusion. Encephalopathy symptoms resolved on the third day of admission while headaches improved on the sixth day of admission. The patient was able to be discharged home.

**Figure 1:**
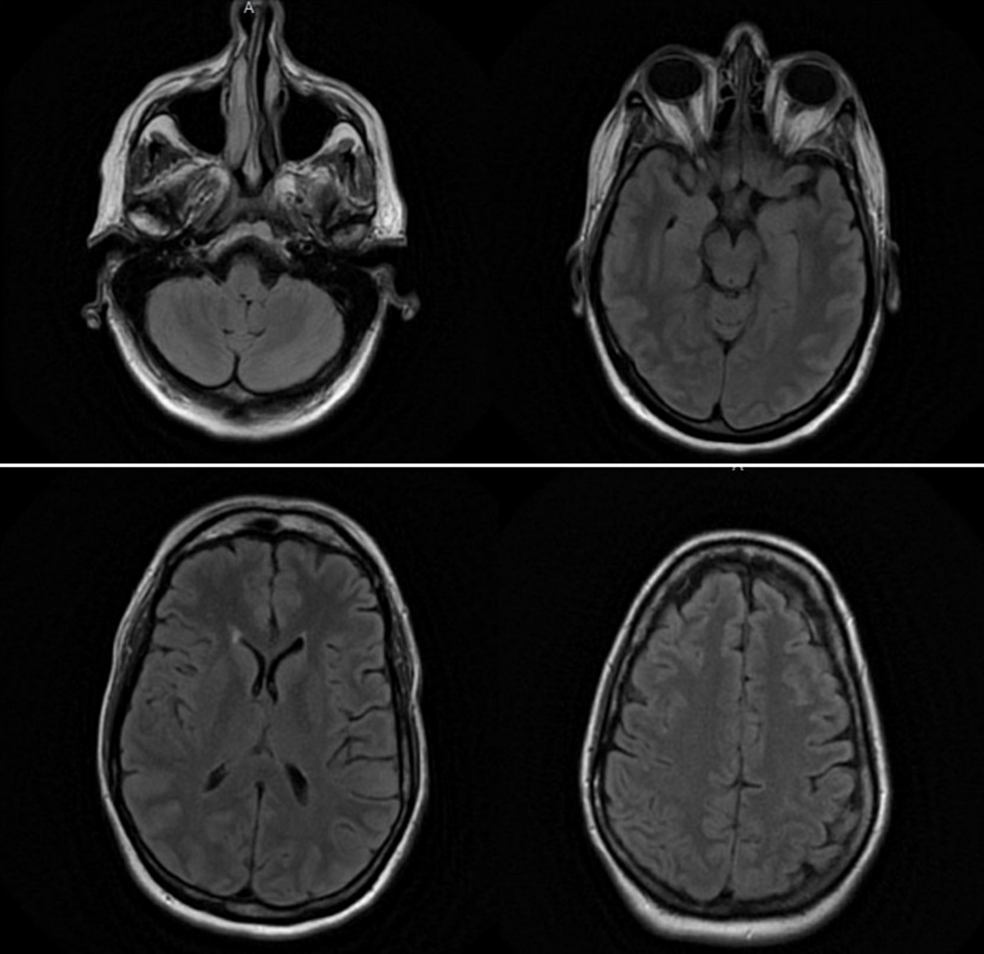
Axial FLAIR MRI demonstrating normal findings. FLAIR: Fluid-attenuated inversion recovery.

Eight days after discharge, the patient presented again to the ER with complaints of general weakness, headache, neck pain, photophobia, bilateral hand paresthesia, and nausea. Physical examination showed an oral temperature of 98.2°F, pulse of 109 bpm, respiratory rate of 18 pm, blood pressure 125/80 mmHg, and oxygen saturation 98% on room air. Laboratory values were WBC 6.3 K/mcL, Hgb 14.7 g/dL, Hct 44.9%, and creatinine 0.9 mg/dL. Electrolytes and UA were normal. UDS was again positive for methadone. Brain CT Scan demonstrated normal findings. MRI of the brain was negative for acute hemorrhage, ischemia, edema, masses, or enhancing lesions (Figure 1). Cervical spine MRI was positive for multiple disc protrusions and moderate spinal stenosis. During this evaluation, the CSF analysis revealed WBC 1,344/cm³ with neutrophil predominance, neutrophils 90%, lymphocyte 10%, red cell count 0/cm³, glucose 47 mg/dL, and protein 122 mg/dL. VDRL was non-reactive. These results were now reflective of bacterial meningitis with significant elevation in the WBC with neutrophil predominance. The patient was started on empirical antibiotic therapy with IV ceftriaxone, vancomycin, ampicillin, and acyclovir.

CSF nucleic acid amplification test (NAAT) was negative for HSV 1 & 2, cytomegalovirus (CMV), enterovirus, human herpesvirus 6 (HHV-6), human parechovirus, varicella-zoster virus, *Cryptococcus neoformans/gattii*, *Escherichia coli*, *Haemophilus influenzae*, *Listeria monocytogenes*, *Neisseria meningitidis*, *Streptococcus agalactiae/pneumoniae.* West Nile Virus IgM/IgG from CSF was also negative. Serology for HIV including HIV 1 & 2 antibodies and HIV 1 p24 antigen were negative. CSF and blood cultures were negative for bacterial and fungal growth. QuantiFERON-TB Gold In-Tube (QFT-GIT) assay was negative. Upon further questioning, the patient reported recently resuming TMP-SMX at home for a right foot ulcer infection. The patient recovered within four days after discontinuation of TMP-SMX. Intravenous antibiotics and acyclovir were discontinued, with complete resolution of symptoms. Symptoms were attributed to the TMP-SMX due to the time of antibiotic initiation, negative CSF cultures, and improvement of symptoms several days after discontinuation of the offending agent.

## Discussion

DIAM continues to be a rare presentation. The first case of TMP-SMX-induced aseptic meningitis (TSIAM) was described in 1983 [6]. Since then, around 41 cases have been reported [2]. It is important to understand that DIAM is a diagnosis of exclusion and a complete medical history that includes a detailed drug history is crucial for diagnosis. Infectious etiology should always be excluded. Bacterial, viral, and fungal pathogens are most commonly associated, but autoimmune disease, malignancy, and less typical infections like spirochetes, mycobacteria, and parasites have also been reported in literature [1]. Symptoms generally include fever, neck stiffness, headache, photophobia, nausea, vomiting, and confusion [8]. In some cases, life-threatening presentations like seizures, hypotension, and respiratory failure have been documented [1, 9]. CSF analysis may be indistinguishable from bacterial meningitis and empirical therapy with IV antibiotics and antiviral is warranted while awaiting cultures.

Little is known about the pathogenesis of DIAM; however, many experts have proposed an immune hypersensitivity reaction [2], which may occur upon initial exposure to an offending agent and without a history of allergy. A history of previously diagnosed DIAM should raise awareness as relapses are common. Management consists of the withdrawal of the causative agent. Many clinicians will continue antibiotic therapy despite negative CSF cultures as sometimes clinical presentation can mimic partially treated bacterial meningitis [9]. Typically, patients with DIAM will have CSF analysis consistent with neutrophilic pleocytosis, identical in most cases to bacterial meningitis [3]. In contrast, the patient's initial CSF analysis showed lymphocyte pleocytosis, hence the initial management with acyclovir during the first encounter. Subsequent viral CSF NAA and cultures were negative. During her second evaluation, CSF analysis demonstrated neutrophilic pleocytosis, suggesting bacterial meningitis (Table 1). In our patient, infectious etiology was excluded, and after discontinuation of TMP-SMX, empirical antibiotic and antiviral therapies were discontinued, with full resolution of symptoms. The patient did not have a history of allergies to TMP-SMX or sulfa drugs.

**Table 1:** CSF analysis comparison. CSF: cerebrospinal fluid.

	Initial CSF Analysis	Second CSF Analysis
Appearance	Clear	Clear
Color	Colorless	Colorless
Red Blood Cells/mm3	0	0
White Blood Cells/mm3	13	1,344
Neutrophils (%)	8	90
Lymphocytes (%)	92	10
Glucose (mg/dL)	55	47
Proteins (mg/dL)	41	122
Cultures	Negative	Negative

## Conclusions

DIAM is still a rare but potentially life-threatening condition that continues to represent a diagnostic challenge for many clinicians. A high level of suspicion and obtaining a detailed history are important for the diagnosis of DIAM. The exclusion of bacterial meningitis is fundamental, and empirical therapy with antibiotics and antiviral agents should be initiated. It is important to understand that a considerable number of other antibiotics have been associated with DIAM to a lesser extent than TMP-SMX. Despite the clinical symptomatology resembling infectious etiologies, withdrawal of the offending agent along with supportive measures are the basis for treatment required in most cases. Prognosis is generally good. This case reflects the importance of obtaining a thorough history and physical to include medication history. 
